# Reducing barriers to post-9/11 veterans’ use of programs and services as they transition to civilian life

**DOI:** 10.1186/s12913-020-05320-4

**Published:** 2020-06-10

**Authors:** Nicole R. Morgan, Keith R. Aronson, Daniel F. Perkins, Julia A. Bleser, Katie Davenport, Dawne Vogt, Laurel A. Copeland, Erin P. Finley, Cynthia L. Gilman

**Affiliations:** 1 Clearinghouse for Military Family Readiness at Penn State University, 114J Henderson Building, University Park, PA 16802 USA; 2 Social Science Research Institute at Penn State University, University Park, USA; 3 Department of Biobehavioral Health, Penn State University, University Park, USA; 4 College of Agricultural Sciences, Department of Agricultural Economics, Sociology and Education, Penn State University, University Park, USA; 5 Women’s Health Sciences Division, National Center for PTSD VA Boston Healthcare System, Boston, USA; 6 Department of Psychiatry, Boston University School of Medicine, Boston, USA; 7 VA Central Western Massachusetts Healthcare System, Leeds, USA; 8 University of Massachusetts Medical School, Quantitative Health Sciences, Worcester, USA; 9 Department of Psychiatry, UT Health San Antonio, San Antonio, USA; 10 Veterans Evidence-based Research Dissemination and Implementation Center, South Texas Veterans Health Care System, San Antonio, USA; 11 Departments of Medicine and Psychiatry, UT Health San Antonio, San Antonio, USA; 12 The Henry M. Jackson Foundation for the Advancement of Military Medicine, Inc., Bethesda, USA

**Keywords:** Veterans, Veteran reintegration, Barrier reduction, Help-seeking stigma, Mental health stigma, Program reach, Program sustainability

## Abstract

**Background:**

Numerous programs exist to support veterans in their transitions to civilian life. Programs are offered by a host of governmental and non-governmental stakeholders. Veterans report encountering many barriers to program participation. This study identified barrier reduction strategies offered by programs that new post-9/11 veterans reported using, determined which strategies veterans use and value, and examined veteran characteristics that impact their odds of using programs that offer barrier reduction strategies.

**Method:**

This study reflects findings from the first wave of data collection of The Veterans Metrics Initiative (TVMI), a longitudinal study examining the military-to-civilian reintegration of new post-9/11 veterans. The websites of programs used by respondents were coded for barrier reduction components. Veterans also indicated which barrier reduction components they found most helpful in meeting their reintegration goals.

**Results:**

Of 9566 veterans who participated in Wave 1 data collection, 84% reported using a program that offered at least one barrier reduction component. Barrier reduction components included tangible supports (e.g., scholarships, cash), increased access to programs, decreased stigma, and encouraged motivation to change. Although only 4% of programs that were used by veterans focused on helping them obtain Veterans Administration benefits, nearly 60% of veterans reported that this component was helpful in reaching their goals. Access assistance to other resources and supports was also reported as a helpful barrier reduction component. For instance, approximately 20% of veterans nominated programs that offered transportation. The study also found evidence of a misalignment between the kinds of barrier reduction components veterans valued and those which programs offered. Veterans from the most junior enlisted ranks, who are at most risk, were less likely than those from other ranks to use barrier reduction components. Study limitations and ideas for future research are discussed.

**Conclusions:**

Despite the evidence that barrier reduction components enhance access to programs and contribute to program sustainability, many programs used by post-9/11 veterans do not offer them. There was also a misalignment between the barrier reduction strategies that veterans value and the strategies offered by programs. Veteran serving organizations should increasingly implement barrier reduction strategies valued by veterans.

## Contributions to the literature

Very little is known about how the transition from active duty service to civilian life unfolds among post-9/11 veterans. While a plethora of programs and supports are offered to veterans, many report difficulties accessing them.

To increase the penetration and reach of programs and services targeted to veterans, barrier reduction efforts are needed, and they must align with what veterans’ report valuing in reaching their reintegration goals.

This study demonstrated that barrier reduction efforts are recognized and needed by veterans, but they are somewhat misaligned with what veterans need, are generally under-used, and special efforts need to be made to target barrier reduction to veterans from the most junior enlisted ranks.

## Background

Currently, there are 2.6 million post-9/11 military veterans in the United States. This population is projected to grow to 3.5 million by 2019 [1]. The majority of these veterans do not experience ongoing adjustment or reintegration problems and make a successful transition back to their communities [2]. However, a substantial minority report struggles with reintegration. Difficult transitions from the military to civilian life have the potential to place a tremendous burden on veterans and their families, communities, and healthcare systems. The reintegration experiences of post-9/11 veterans appear to be impacted by a variety of factors operating at different ecological levels (i.e., individual, interpersonal, community, and societal) and to vary from person to person [3].

Some of the most common challenges experienced by post-9/11 veterans occur within four domains of well-being: [1] vocational (i.e., employment and education) [2]; legal, financial, and housing [3]; health; and [4] social relationships. In terms of vocational challenges, many post-9/11 veterans do not have a job when they leave the military [4–6]. Young post-9/11 veterans appear to have the most employment problems [7]. Moreover, veterans have reported challenges understanding and utilizing their Post-9/11 GI Bill benefits [8]. Furthermore, while veterans account for only 6% of the U.S. population, they comprise 10% of those without stable housing [9]. Some veterans live with health problems such as brain or musculoskeletal injuries, burns, or limb loss [10], and 32% of post-9/11 veterans have a service-connected disability [11]. Common mental health problems include post-traumatic stress disorder (PTSD), depression, and suicidality [12, 13]. In addition, post-9/11 veterans are vulnerable to social support deficiencies because of differences in cultural norms between military and civilian communities [14]. Difficulties in making and sustaining family connections can also occur, including marital strain, role confusion, and re-connecting with children [15].

### A typology of barriers to programs and services

Despite the well-documented needs of post-9/11 veterans and the plethora of programs available to them [16], many veterans do not seek out or use veteran programs or services [17]. There are a number of real and perceived barriers to accessing these supports. A recent study indicated there were four primary reasons new veterans reported not using programs during the early military to civilian transition period [17]. These included the following: (a) not needing assistance, (b) not identifying a program or service that sufficiently met their needs, (c) not understanding the kinds of programs for which they qualified; and (d) not knowing where to obtain support and assistance. The reasons for program non-use reported were not surprising given that veterans frequently lack awareness of community-based supports and services [3, 18], and they do not always understand their U.S. Department of Veterans Affairs (VA) benefits [19].

Currently, there are over 40,000 programs in the United States purporting to assist veterans with transitioning to civilian life [20]. Veterans report having difficulty discerning which, if any, of these programs are relevant to them or whether they qualify to use these programs [21]. Navigating the VA system can also be daunting for veterans [22]. Occasional extended waiting times for appointments; lengthy paperwork; and difficulty navigating the healthcare system, whether in person or online, have been found to reduce the likelihood that service members will seek needed care [23, 24]. In order to address the prevalence of low utilization, programs and services are employing components that can help reduce barriers to care and services at VA, private, and community-based support programs.

### Barrier reduction components

Theory and research in the area of implementation and dissemination science indicates that effective program implementation requires four essential elements: [1] relevant content, [2] a process for teaching the content, [3] approaches for sustaining the program over time, and [4] barrier reduction strategies [25, 26]. Barrier reduction components may assist veterans in accessing the support they need to make more successful transitions to civilian life [27]. These components increase program participation by providing tangible supports, improving program access, and helping people make intra-individual changes to reduce help seeking stigma and increase motivation for change.

*Tangible supports* are the physical resources, assistance, and/or monetary supports provided to veterans to directly assist them in meeting their basic and higher-order needs [28, 29]. To reduce individual-level and family-level barriers to care, programs have helped address veterans’ basic needs (e.g., provide food, shelter, and/or housing accessibility modifications). Programs have also addressed higher-order needs by providing direct financial support for education in the form of offering scholarships and money for books, providing legal advice, and giving veterans information regarding strategies about how to access benefits. VA educational benefits, particularly the Post-9/11 GI Bill, and the VA Home Loan Program are important and widely used tangible supports because they address two common challenges experienced by veterans reintegrating into civilian life – education and housing [28, 29]. The Post-9/11 GI Bill of 2008 provides for tuition and fees, a housing allowance, books and supplies stipend, and a one-time rural benefit (relocation stipend of $500). The VA assists veterans and eligible surviving spouses become homeowners by providing direct home loans and a VA-backed home loan guaranty, which typically loosens the requirements a number of lenders have and makes it easier for veterans to obtain mortgages.

*Program access components* typically provide logistical supports that make it easier for veterans to engage with programs. The VA healthcare system is offered at no cost for veterans, which is a critical program access barrier reduction component. Transportation is also sometimes provided by the VA to veterans who live far from a VA clinic, which makes it easier for them to attend appointments [30]. In addition, the VA provides virtual services to increase access, such as telemedicine options [31, 32]. Outside of the VA system, some veteran programs offer child care, so parents/guardians can fully participate in the program (e.g., Yellow Ribbon Reintegration Program). Other programs offer free healthcare or provide services on a sliding fee scale (e.g., Volunteers in Medicine).

*Intra-individual change* component involves: [1] promoting intrinsic motivation to obtain assistance; and/or [2] decreasing help-seeking stigma (Morgan et al., 2018). Intra-individual change components provide an opportunity to initiate the behavior with little costs (e.g., free month of a gym membership) thereby promoting intrinsic motivation to change. Help-seeking stigma is common among veterans [33]. Veterans report perceiving that people in society hold unfavorable beliefs towards those who have or seek treatment for mental health problems, which, in turn, deters help-seeking [34]. Efforts to combat veteran help-seeking exist (e.g., Real Warrior Campaign, Buddy-to-Buddy). The Real Warrior Campaign is a multimedia public awareness effort. Buddy-to-Buddy uses peer educators [35] and contact strategies [36–38] to decrease help-seeking stigma.

### Current study

To our knowledge, this is the first study to describe the barrier reduction components that veterans encounter in the VA and non-VA programs that they use. Extant research has focused primarily on barrier reduction related to the use of VA mental healthcare. Many veterans use non-VA programs in support of employment, education, financial, health, and social goals [16]. Recently discharged veterans were asked to report which programs or services they used. Then, they provided detailed information on two of the programs they used. Finally, they were asked which of the types of barrier reduction components they used to help them achieve their goals. The percentage of barrier reduction components veterans used to achieve their goals was calculated. Second, through web-based coding, the percentage of barrier reduction components offered to the veteran was calculated. Third, among the barrier reduction components offered, the proportion of veterans who used those components was identified. Finally, exploratory analysis investigated subgroup difference in the use of programs that contain barrier reduction components.

## Methods

### Participants

The population of veterans who had separated from active duty service or de-activated from active duty status in the prior 4 months between June–September 2016 were identified from Veterans Affairs and Department of Defense Identity Repository (VADIR) and were invited to participate in the study (*n* = 48,965). Detailed information on the sample, procedures, and participant demographics have been published elsewhere [39]. Complete data was obtained from nearly 20% of the population (*n* = 9566). The majority of the veterans were male 82% (*n* = 6734), White, Non-Hispanic 64% (*n* = 5215), and from the enlisted ranks (77%).

### Identifying barrier reduction components

Veterans were asked 37 questions about programs they had used in each of four well-being domains: [1] vocational (employment and education [2]; legal, financial, and housing [3]; health (physical and mental); and [4] social/personal relationships. Additional questions were asked about their current use of educational programs through the Post-9/11 and Montgomer GI Bills, the VA home loan program, and VA hospitals, clinics, and Vet Centers. Veterans were then also asked to provide in-depth information on up to two of the programs they had used [1].

To identify the barrier reduction components within those programs, respondents were asked several additional specific questions. First, they were asked if any of the following types of support from each program they had used helped them achieve their goals: scholarship for education, cash, housing (including accessibility modifications), food, clothes or other physical objects, legal advice, access to benefits, among others. Second, veterans were asked if any of the programs they used made it easier to access the program by providing transportation, insurance, child care, stigma reduction strategies or messaging, bolstering motivation to change, and others. Finally, veterans were asked the extent to which barrier reduction strategies helped them achieve their reintegration goals.

#### Procedures

### Identification of program barrier reduction components

A list of barrier reduction components was derived from a review of the research literature using grounded theory. Rotheram-Borus et al. (2009) provided a framework specifically for barrier reduction components [25]. Veterans were asked to provide the names of programs they had used since separating from the military. The websites of programs that were nominated by three or more veterans were examined to determine which barrier reduction components were offered (i.e.., tangible supports, program access, intra-individual change*)*. The websites were coded for the presence of barrier reduction components using a distilling process [26]. Tangible support components included scholarships for education, monetary gifts, and discounted pricing on goods and services. Barrier reduction components related to access assistance included having program content available online, a sliding scale fee structure, and available child care. Intra-individual change components focused on increasing motivation to change and reducing help-seeking stigma.

Coding was conducted using qualitative software NVivo 12 (QSR International, 2016). Two independent raters coded programs by indicating whether each component was either present or absent for each program. Coders applied all relevant codes to a program; therefore, codes were not mutually exclusive. Reliability among coders was established by having a third *expert* coder check coding consistency, discussing discrepancies, and coming to agreement on the final codes and definitions of each component.

### Data analytic approach

In survey studies that do not use random sampling, there is always a question regarding the degree to which the responses may be biased because participants in the study may not fully represent the population from which the sample was drawn [40]. In the current study, three variables were available to apply weights (i.e., gender, branch, and paygrade). Differences between the weighted and unweighted proportion estimates were examined for design effects. A design effect ratio is used to indicate if the sampling variability for each estimate was increased or decreased by the design used. When a design effect ratio is greater than one, a larger sample would have needed to be drawn in order to have the precision as a random sample. A design effect ratio less than 1 indicates that fewer cases would be needed to obtain the results of a random sample [41]. No meaningful design effects were found suggesting that the current study sample was representative of the three known characteristics (i.e, gender, paygrade, branch) of this population of post-9/11 veterans who transitioned between June–September 2016. Thus, weighted proportion estimates were computed using STATA svy: proportion; or svy: logistic (StataCorp, 2013). Logistic regression analyses were used to estimate the odds ratios of barrier reduction component use as a function of several specific subgroup classifications (e.g., gender, branch, pay grade, combat exposure).

## Results

### The use of VA programs as a barrier reduction component

The vast majority of veterans (84%; *n* = 8010) reported using at least one program or VA benefit. Approximately, three-quarters of the respondents nominated a VA benefit. Nearly half (43%) of post-9/11 veterans reported using educational benefits. Approximately 34% used the Post-9/11 GI Bill, while 9% used the Montgomery GI Bill, and 32% of veterans used the VA home loan program. Nearly 35% of veterans used VA hospitals, clinics, and/or Vet Centers.

### Barrier reduction components coded, reported, and alignment of offered-used components

Program websites were coded for 91% of all the nominations (i.e., programs nominated by three or more veterans), which resulted in 656 unique programs being coded. In addition, 239 programs were identified by coders from nominations. However, these only included a program type (e.g., scholarship) not a specific program that was able to be identified via a web search.

Table 1 provides a summary of the weighted proportions of the following: (a) programs that were coded for barrier reduction components (*n* = 895); (b) veterans who self-reported using a barrier reduction component that helped them achieve their goals (*n* = 4308); and (c) the alignment among programs that offered a program component and the proportion of veterans who self-reported using it.

**Table 1:** Barrier Reduction Components Coded, Helpfulness in Achieving Goals, Alignment of Coding and Self-Report

	% of Programs Coded	Helpful to Achieve Goals (*n* = 4308)		Alignment of Programs Offered and Used	
	(n = 895)	% (SE)	DE	% (SE)	DE
Tangible Support Components	48.6%	48.2% (0.8%)	1.17	51.6% (1.0%)	1.17
Assistance in obtaining VA benefits	NC	23.7% (0.7%)	1.17	–	–
Non-VA tuition discounts/scholarships	17.5%	12.2% (0.6%)	1.31	32.4% (1.7%)	1.24
Cash	14.5%	5.1% (0.4%)	1.33	10.7% (1.3%)	1.25
Clothing & other consumer goods	11.1%	2.3% (0.3%)	1.24	5.9% (1.1%)	1.32
Discounted pricing on goods/services	10.4%	Not Mentioned		Not applicable	
Legal advice	10.1%	6.5% (0.4%)	1.19	16.5% (2.3%)	1.38
Housing or accessibility modifications	6.9%	5.4% (0.4%)	1.25	12.0% (1.4%)	1.18
Job placement services	5.6%	0.2% (0.1%)	1.02	1.3% (1.3%)	1.69
Licensing Assistance	5.4%	0.6% (0.1%)	1.11	9.2% (2.8%)	1.21
Food	5.0%	3.1% (0.3%)	1.25	16.1% (2.4%)	1.29
Veterans Representative	4.6%	Not Mentioned		Not applicable	
“Other” Veteran benefits	3.4%	Not Mentioned		Not applicable	
Free admission to arts/entertainment	2.9%	Not Mentioned		Not applicable	
Program Access Components ^a^	30.6%	21.5% (0.7%)	1.16	21.3% (1.3%)	1.15
Content available online ^b^	94.4%	Not Mentioned		Not applicable	
Provided transportation/ close proximity	16.0%	2.7% (0.3%)	1.22	4.3% (0.8%)	1.31
Payed fees	5.0%	Not Mentioned		Not applicable	
Non-VA insurance/free medical care	5.0%	15.6% (0.06)	1.17	12.6% (2.2%)	1.21
Offered lodging	5.3%	Not Mentioned		Not applicable	
Provided child care	3.2%	0.9% (0.1%)	1.12	5.1% (2.3%)	1.05
Intrinsic Components	9.0%	19.0% (0.6%)	1.18	32.6% (2.7%)	1.16
Increased motivation to change	6.3%	17.5% (0.6%)	1.18	29.3% (3.7%)	1.17
Reduced stigma	2.7%	4.9% (0.3%)	1.10	14.1% (2.9%)	1.12

Note: Weighted Estimates; *SE* Standard Error, *DE* Design Effect, ^a^ = excluding content available online ^b^ = Among programs with a URL website

Fifty-six percent of programs offered a tangible support component. The first column of Table 1 represents the proportion of programs that were coded for barrier reduction components. As can be seen, the most frequently coded barrier reduction component was content available online (94.4%, program access). The proportions of the other barrier reduction components coded were significantly lower and ranged from a high of 17.5% (other tuition discounts or scholarship, tangible) to a low of 2.7% (stigma reduction, intra-individual change). Indeed, 13 of the 21 components were coded as being present less than 10% the time. Lastly, no programs were coded as helping for obtaining VA benefits (e.g., Veterans of Foreign Wars volunteers).

The second column represents the self-report of veterans who nominated at least one program to describe in detail and who used a barrier reduction component and reported that the component helped them achieve their goals. The most commonly mentioned barrier reduction components that helped veterans reach their goals were assistance for obtaining VA benefits (23.7%, tangible), increasing motivation to change (17.5%, intra-individual change), provision of non-VA insurance or free medical care (15.6%, program access), and non-VA tuition discounts or scholarships (12.2%, tangible). The remainder of barrier reduction components were not or were rarely mentioned as being used and having helped veterans achieve their goals.

The final column in Table 1 reports that, among programs that provided a barrier reduction component, the proportion of veterans who reported using that component varied widely. The most frequently aligned components were other tuition discounts or scholarships (32.4%), increasing motivation to change (29.3%), and assistance for obtaining VA benefits (28%). The alignment of the other barrier reduction components with veteran use of those components ranged from 16.5% for legal advice to 1.3% for job placement services.

Looking across the columns of Table 1, several findings warrant highlighting. First, while having content available online (program access) was coded as being present in 94.4% of programs, it was not mentioned by veterans as being used or having helped them reach their goals. Second, assistance for obtaining VA benefits was not coded as being present for any nominated programs but was mentioned as being used by and helping to achieve the goals of 23.7% of veterans. Third, the majority of coded barrier reduction components were neither used nor viewed as being helpful by veterans. For example, 16% of programs provided transportation, however, only 2.7% of veterans reported using or benefitting from programs that provided transportation. Similarly, 14.5% of nominated programs assisted veterans by providing them with emergency funds (i.e., cash), only 5.1% of veterans used or benefited from these programs. Finally, for a number of components, there was little consistency among the coded components, their use and perceived helpfulness, and the alignment between a component’s presence and its reported use. For example, only 6.3% of programs included a component to increase motivation, however, 29.3% of veterans reported using programs with that component and 17.5% of veterans reported benefiting from the increased motivation component. Thus, it appears that veterans value this component and find that it helped them in their transition to civilian life.

### Predictors of the use of programs with barrier reduction components

VA-sponsored tangible supports included the Montgomery GI Bill or Post-9/11 GI Bill; VA home loan program; and use of VA hospitals, clinics, or Vet Centers. Veterans from the Air Force were less likely to utilize any GI Bill compared to Army veterans; however, veterans from the National Guard or Reserve were two times more likely to utilize the GI Bill than veterans from the Army (see Table 2). In addition, current National Guard and Reserve members were 40% more likely to utilize a GI Bill. Officers were less likely to utilize any GI Bill compared to veterans from the junior enlisted ranks (E1 to E4), while veterans from the senior enlisted ranks (E5 to E6) were 20% more likely to utilize any GI Bill compared to junior enlisted veterans.

**Table 2:** Who Is More Likely to Use VA Tangible Support Components?

	GI Bill Odds Ratio [CI]	VA home loan Odds Ratio [CI]	VA hospital, clinic, or Vet Center Odds Ratio [CI]
Constant			
Army (ref)			
Navy	0.94 [0.82, 1.07]	0.94 [0.81, 1.09]	0.81 [0.70, 0.93]***
Air Force	0.86 [0.75, 0.99]*	1.24 [1.07, 1.44]***	0.77 [0.66, 0.89]***
Marine Corps	0.99 [0.86, 1.13]	0.60 [0.51, 0.70]***	1.03 [0.89, 1.20]
National Guard or Reserve	2.19 [1.25, 3.83]*	0.51 [0.26, 1.00]	1.20 [0.67, 2.14]
Male	1.01 [0.89, 1.14]	1.19 [1.03, 1.37]*	1.13 [0.98, 1.30]
E1 to E4 (ref)			
E5 to E6	1.21 [1.08, 1.36]***	3.06 [2.65, 3.53]***	1.18 [1.04, 1.34]*
E7 to E9	0.95 [0.82, 1.10]	7.14 [6.02, 8.46]***	1.11 [0.94, 1.30]
W1 to W5	1.04 [0.70, 1.54]	8.87 [5.89, 13.35]***	1.49 [1.00, 2.21]
O1 to O3	0.69 [0.57, 0.82]***	4.23 [3.49, 5.14]***	0.91 [0.74, 1.13]
O4 to O7	0.65 [0.54, 0.77]***	7.08 [5.88, 8.51]***	0.91 [0.75, 1.09]
Currently NGR after AD	1.39 [1.22, 1.59]***	0.84 [0.73, 0.97]*	0.77 [0.66, 0.90]***
Currently serving NGR	0.86 [0.49, 1.53]	1.37 [0.70, 2.70]	0.44 [0.24, 0.80]*
Service support occupation (ref)			
Combat arms occupation	1.18 [1.04, 1.35]*	0.84 [0.73, 0.96]*	0.97 [0.84, 1.11]
Combat support occupation	1.19 [1.07, 1.32]***	0.98 [0.88, 1.11]	1.05 [0.94, 1.18]
Warfare exposure	1.07 [0.96, 1.19]	1.47 [1.31, 1.65]***	1.42 [1.26, 1.60]***
Medical discharge	0.99 [0.90, 1.08]	1.06 [0.96, 1.18]	1.24 [1.11, 1.38]***
White, Non-Hispanic (ref)			
Black NH	1.16 [0.99, 1.35]	0.69 [0.58, 0.81]***	1.33 [1.13, 1.57]***
Hispanic	0.98 [0.85, 1.11]	0.84 [0.72, 0.98]*	1.34 [1.16, 1.55]***
Asian, HPI NH	1.02 [0.82, 1.28]	1.21 [0.93, 1.58]	1.62 [1.27, 2.06]***
More than one race NH	1.09 [0.88, 1.34]	0.91 [0.71, 1.15]	0.85 [0.67, 1.08]
Other race not listed NH	0.73 [0.49, 1.09]	2.18 [1.25, 3.82]*	1.58 [0.96, 2.60]
Ongoing physical health condition	1.02 [0.92, 1.14]	1.18 [1.05, 1.33]*	2.33 [2.07, 2.63]***
Ongoing mental health condition	1.02 [0.90, 1.17]	1.13 [0.97, 1.31]	1.58 [1.38, 1.81]***
Screened positive PTSD symptoms	1.00 [0.88, 1.14]	1.02 [0.88, 1.17]	1.12 [0.97, 1.28]
Probable alcohol abuse	0.94 [0.86, 1.04]	0.83 [0.75, 0.93]***	0.97 [0.87, 1.08]
Probable depression	0.67 [0.57, 0.78]***	0.86 [0.73, 1.02]	1.15 [0.98, 1.34]
Probable anxiety	1.08 [0.94, 1.25]	0.99 [0.84, 1.16]	1.05 [0.91, 1.23]
Full-time employment	0.47 [0.43, 0.52]***	1.66 [1.49, 1.84]***	0.63 [0.56, 0.70]***

^*^p < .05; ^**^p < .01; ^***^p < .001; (*n* = 9466; population size = 48,427); *ref.* reference group, *AD* Active Duty, *NGR* National Guard/ Reserve, *HPI* Hawaiian Pacific Islander, *NH* Non-Hispanic, *CI* confidence interval

Male veterans were 20% more likely to utilize the VA home loan program. All senior enlisted ranks and officer ranks were more likely than junior enlisted veterans to utilize the VA home loan program. Veterans exposed to combat were 50% more likely to utilize the VA home loan program. In general, minority veterans were less likely to utilize the VA home loan program compared to White, Non-Hispanic veterans. However, veterans from other racial groups were two times more likely to utilize the VA home loan program compared to White, Non-Hispanic veterans. Veterans who screened positive for probable alcohol abuse were less likely to utilize the VA home loan program. Veterans who had full-time employment were 66% more likely to utilize the VA home loan program.

Veterans from the Navy and Air Force and National Guard and Reserve members were less likely to utilize the VA healthcare system compared to veterans from the Army. Veterans who were exposed to combat, medically discharged, and had ongoing physical and mental health conditions were more likely to utilize the VA healthcare system. Veterans who were from ethnic minority groups (i.e., Black, Non-Hispanic; Hispanic; and Asian, Non-Hispanic) were all more likely to utilize the VA healthcare system compared to White, Non-Hispanic veterans. Veterans working full-time were less likely to utilize the VA healthcare system.

Additional analysis was conducted on the sub-sample of veterans who reported using tangible support components outside of the VA (see Table 3). Veterans from the Marine Corps were 57% more likely than those from the Army, and male veterans were 58% more likely to use non-VA scholarships. Veterans from the Navy and Marine Corps were both 75% more likely to use cash allowances than their Army peers. Compared to veterans from the most junior enlisted ranks, those from most of the higher ranks were significantly less likely to obtain cash assistance.

**Table 3:** Who Is More Likely to Use Non-VA Tangible Supports?

	Other Scholarship Odds Ratio [CI]	Cash (non-tuition support) Odds Ratio [CI]	Housing Odds Ratio [CI]	Legal advice Odds Ratio [CI]
Constant				
Army (ref)				
Navy	1.28 [0.92, 1.77]	1.75 [1.13, 2.72] *	1.09 [0.69, 1.70]	1.30 [0.89, 1.91]
Air Force	1.07 [0.76, 1.51]	0.85 [0.49, 1.49]	1.17 [0.75, 1.83]	1.44 [0.99, 2.09]
Marine Corps	1.57 [1.14, 2.16]*	1.75 [1.09, 2.81] *	1.32 [0.84, 2.06]	1.02 [0.66, 1.58]
National Guard or Reserve	1.14 [0.26, 4.91]	1.37 [0.16, 12.01]	2.13 [0.49, 9.14]	0.88 [0.11, 7.12]
Male	1.58 [1.16, 2.15]***	0.97 [0.64, 1.47]	1.57 [1.03, 2.41] *	1.03 [0.72, 1.49]
E1 to E4 (ref)				
E5 to E6	0.71 [0.54, 0.93]*	0.55 [0.37, 0.82] ***	0.63 [0.41, 0.95] *	0.69 [0.47, 1.01]
E7 to E9	0.57 [0.40, 0.81]***	0.43 [0.26, 0.71] ***	0.40 [0.24, 0.67] ***	0.72 [0.46, 1.14]
W1 to W5	0.32 [0.10, 1.10]	0.34 [0.10, 1.20]	0.30 [0.09, 1.05] *	0.59 [0.22, 1.60]
O1 to O3	0.48 [0.32, 0.73]***	0.36 [0.18, 0.70] ***	0.92 [0.52, 1.61]	0.48 [0.25, 0.90]*
O4 to O7	0.30 [0.19, 0.46]***	0.28 [0.15, 0.54] ***	0.51 [0.30, 0.87] *	0.93 [0.58, 1.46]
Currently NGR after AD	1.40 [1.03, 1.89]*	1.27 [0.81, 2.01]	0.71 [0.45, 1.11]	0.93 [0.62, 1.39]
Warfare exposure	1.01 [0.78, 1.31]	1.44 [1.01, 2.05]	1.35 [0.93, 1.96]	1.20 [0.87, 1.66]
White, Non-Hispanic (ref)				
Black NH	0.81 [0.56, 1.19]	1.05 [0.63, 1.76]	1.25 [0.77, 2.03]	1.00 [0.62, 1.61]
Hispanic	0.89 [0.65, 1.22]	1.45 [0.94, 2.23]	1.01 [0.64, 1.59]	1.59 [1.08, 2.33]*
Asian, HPI NH	0.89 [0.52, 1.52]	1.52 [0.75, 3.11]	1.60 [0.82, 3.10]	1.00 [0.47, 2.13]
More than one race, NH	1.34 [0.86, 2.10]	1.38 [0.73, 2.63]	1.54 [0.84, 2.82]	2.28 [1.40, 3.69]***
Medical discharge	0.98 [0.79, 1.22]	0.86 [0.60, 1.23]	1.09 [0.78, 1.53]	1.17 [0.85, 1.62]
Ongoing physical health condition	0.98 [0.76, 1.25]	1.00 [0.67, 1.49]	0.98 [0.67, 1.44]	1.10 [0.77, 1.58]
Ongoing mental health or emotional condition	1.05 [0.78, 1.42]	1.42 [0.94, 2.12]	1.10 [0.73, 1.65]	1.16 [0.80, 1.70]
Screened positive PTSD symptoms	0.88 [0.66, 1.18]	0.98 [0.67, 1.44]	1.23 [0.84, 1.77]	1.85 [1.25, 2.75]***
Full-time employment	0.53 [0.41, 0.67]*	0.91 [0.65, 1.29]	0.86 [0.62, 1.19]	0.90 [0.67, 1.21]

^*^p < .05; ^**^p < .01; ^***^p < .001; (*n* = 4267; population size = 19,599); *ref.* reference group, *AD* Active Duty, *NGR* National Guard/ Reserve, *HPI* Hawaiian Pacific Islander, *NH* Non-Hispanic, *CI* confidence interval

Compared to Army veterans, those from the National Guard and Reserves were 57% more likely to use tangible supports related to housing (e.g., mortgage counseling). Relative to veterans from the most junior enlisted ranks, those from most of the higher ranks were significantly less likely to utilize programs that provided a housing benefit. Hispanic veterans and veterans who identified as Non-Hispanic multi-racial were two times more likely to report using programs that provided legal advice compared to White, Non-Hispanic veterans. Finally, veterans with a probable diagnosis of PTSD were 85% more likely to use programs that provided legal advice compared to those without PTSD.

Table 4 describes the characteristics of veterans who used barrier reduction components related to access assistance. Veterans from the Air Force were less likely to report utilizing a program with a transportation component compared to Army veterans. Veterans from the Marine Corps were less likely to utilize programs that provided access to medical care compared to veterans from the Army. Hispanic veterans were 44% more likely to utilize programs that provided access to medical care and aided with access to insurance compared to White, Non-Hispanic veterans. Veterans with probable PTSD symptoms were two times more likely to utilize programs that offered transportation compared to veterans without PTSD symptoms.

**Table 4:** Who Is More Likely to Use Access Components?

	Provided Transportation Odds Ratio [CI]	Provided Insurance or Free Medical Care Odds Ratio [CI]
Constant		
Army (ref)		
Navy	0.56 [0.28, 1.10]	1.10 [0.84, 1.44]
Air Force	0.38 [0.20, 0.72]***	1.21 [0.94, 1.56]
Marine Corps	0.77 [0.42, 1.43]	0.70 [0.51, 0.96] ^*^
National Guard or Reserve	2.87 [0.62, 13.32]	2.06 [0.72, 5.86]
Male	0.76 [0.45, 1.29]	0.99 [0.77, 1.27]
E1 to E4 (ref)		
E5 to E6	0.90 [0.50, 1.62]	1.06 [0.81, 1.39]
E7 to E9	1.11 [0.59, 2.08]	0.91 [0.66, 1.25]
W1 to W5	1.84 [0.56, 6.02]	1.22 [0.60, 2.46]
O1 to O3	0.47 [0.20, 1.15]	1.18 [0.83, 1.67]
O4 to O7	0.81 [0.40, 1.67]	1.24 [0.90, 1.71]
Currently NGR after AD	1.59 [0.92, 2.77]	1.04 [0.80, 1.35]
Warfare exposure	0.86 [0.52, 1.41]	1.07 [0.86, 1.32]
White, Non-Hispanic (ref)		
Black NH	1.40 [0.76, 2.59]	0.77 [0.55, 1.09]
Hispanic	0.66 [0.35, 1.27]	1.44 [1.10, 1.87] ^*^
Asian, HPI NH	0.84 [0.29, 2.44]	0.91 [0.56, 1.49]
More than one race, NH	0.93 [0.39, 2.22]	0.98 [0.65, 1.48]
Medical discharge	1.34 [0.88, 2.03]	0.98 [0.81, 1.20]
Ongoing physical health condition	0.93 [0.52, 1.66]	0.93 [0.75, 1.15]
Ongoing mental health or emotional condition	0.77 [0.42, 1.40]	1.06 [0.81, 1.38]
Screened positive PTSD symptoms	2.08 [1.19, 3.63] ^*^	1.18 [0.90, 1.55]
Full-time employment	0.77 [0.48, 1.23]	1.25 [1.03, 1.52] ^*^

^*^p < .05; ^**^p < .01; ^***^p < .001; (*n* = 4267; population size = 19,599); *ref.* reference group, *AD* Active Duty, *NGR* National Guard/ Reserve, *HPI* Hawaiian Pacific Islander, *NH* Non-Hispanic, *CI* confidence interval

Table 5 describes the characteristics of veterans using barrier reduction components that focused on intra-individual change. Those from the senior enlisted ranks, warrant officers, and officers were 88% to 3 times, respectively, more likely to report using programs that reduced stigma compared to veterans from the junior enlisted ranks. Veterans with an ongoing mental health or emotional condition were two times more likely to utilize programs that reduced stigma than those without those symptoms. Veterans with a probable PTSD diagnosis were 44% more likely to use a program that increased motivation to utilize the program compared to veterans without PTSD.

**Table 5:** Who Is More Likely to Use Intrinsic Components?

	Reduced Stigma Odds Ratio [CI]	Increased Motivation to Change Odds Ratio [CI]
Constant		
Army (ref)		
Navy	0.97 [0.65, 1.46]	1.19 [0.93, 1.51]
Air Force	0.85 [0.54, 1.34]	1.03 [0.80, 1.33]
Marine Corps	1.24 [0.80, 1.94]	1.11 [0.85, 1.47]
National Guard or Reserve	1.13 [0.15, 8.29]	0.56 [0.12, 2.54]
Male	0.96 [0.65, 1.42]	1.05 [0.83, 1.33]
E1 to E4 (ref)		
E5 to E6	1.88 [1.17, 3.01] ^*^	0.87 [0.68, 1.11]
E7 to E9	1.93 [1.13, 3.29] ^*^	0.79 [0.59, 1.07]
W1 to W5	3.07 [1.18, 7.98] ^*^	1.10 [0.59, 2.04]
O1 to O3	2.39 [1.28, 4.46] ^*^	0.91 [0.65, 1.28]
O4 to O7	2.25 [1.25, 4.07] ^*^	0.84 [0.61, 1.16]
Currently NGR after AD	0.94 [0.57, 1.55]	1.08 [0.84, 1.40]
Warfare exposure	1.11 [0.76, 1.62]	1.11 [0.90, 1.38]
White, Non-Hispanic (ref)		
Black NH	1.21 [0.79, 1.87]	1.19 [0.90, 1.58]
Hispanic	1.10 [0.70, 1.72]	1.23 [0.95, 1.58]
Asian, HPI NH	1.17 [0.51, 2.68]	1.23 [0.80, 1.90]
More than one race, NH	0.75 [0.38, 1.46]	1.43 [0.99, 2.07]
Medical discharge	1.16 [0.85, 1.59]	0.95 [0.77, 1.18]
Ongoing physical health condition	1.26 [0.85, 1.86]	1.00 [0.81, 1.24]
Ongoing mental health or emotional condition	2.05 [1.32, 3.17] ^***^	1.04 [0.81, 1.33]
Screened positive PTSD symptoms	1.29 [0.87, 1.92]	1.44 [1.13, 1.82] ^*^
Full-time employment	0.79 [0.56, 1.10]	0.83 [0.68, 1.00]

^*^p < .05; ^**^p < .01; ^***^p < .001; (*n* = 4267; population size = 19,599); *ref.* reference group, *AD* Active Duty, *NGR* National Guard/ Reserve, *HPI* Hawaiian Pacific Islander, *NH* Non-Hispanic, *CI* confidence interval

## Discussion

To our knowledge, this was the first study to examine barrier reduction components present in programs that are designed to assist post-9/11 veterans as they transition from military to civilian life. Perhaps most striking was the finding that the vast majority of programs did not offer any barrier reduction components. This is a problem from practical, theoretical, and policy perspectives. Practically, many post-9/11 veterans report barriers to programs that they need [3, 17–19]. From a theoretical perspective, research in program implementation science has consistently demonstrated that barrier reduction is critical to bolster program participation and sustainability [25, 26]. To be most effective, programs should consider using best practices that have been documented in the field of implementation science. The VA has made advances in using implementation science insights by introducing transportation assistance programs and providing satellite clinics in rural areas, and offering telehealth options. The present study suggests that non-VA programs should invest in barrier reduction strategies as well.

The study also revealed that a low proportion of new post-9/11 veterans report using and benefiting from barrier reduction components. The most commonly mentioned barrier reduction component that was both used and helpful (i.e., assistance with obtaining VA benefits) was mentioned by less than 25% of the sample. Seven components coded from websites were not mentioned by any veterans, and a sizeable number of barrier reduction components were only mentioned by less than 10% of them. The most parsimonious explanation for this finding is that recently separated post-9/11 veterans do not need to use programs or take advantage of barrier reduction components. A number of studies have shown that most veterans make a healthy transition from military life to civilian life [2]. Perhaps, the need for programs and the need for barrier reduction components increase over time. Future studies should examine this question. Nonetheless, a sizeable number of recently separated post-9/11 veterans do not access programs often because they do not understand their eligibility, do not know what programs they qualify for, cannot find an appropriate program, or encounter other challenges to help seeking [17].

From a policy perspective, VA and non-VA program providers should consider enhancing veteran awareness of their programs, providing clear explanations as to eligibility requirements, offering strategic referrals to programs for which veterans are eligible, and prioritizing barrier reduction strategies in their strategic plans. Given limited resources, efforts should be made by programs to include barrier reduction components. For new post-9/11 veterans, the current study suggested that providing assistance with obtaining VA benefits, increasing motivation to change, provision of non-VA insurance or free medical care, and non-VA tuition discounts/ scholarships are the most important barrier reduction components in helping them reach their goals. Unfortunately, very few programs offer these components. For example, while only 6.3% of programs offered an increased motivation to change component, 17.5% of veterans reported using and benefiting from this type of component. Because veterans value the increased motivation to change component, programs should consider adopting this component (e.g., using motivational interviewing). Just as importantly, a low proportion of veterans report using and benefiting from barrier reduction components. Thus, not only are barrier reduction components not offered enough, but the quality and/or impact of these components may be suspect. Indeed, research on the impact of barrier reduction components is lacking, and future research should address this gap. Research in this area would provide needed information for developing policies to assist veterans make healthy transitions to civilian life.

The results of this study also demonstrate that there is often a misalignment among the barrier reduction components offered by programs and the components that veterans reported as being most helpful. For example, while increased motivation to change was coded in only 6.3% of programs, 17.5% of veterans reported using and benefiting from this component. In addition, among programs offering this component, 29.3% of veterans reported using it. While there is no research related to barrier reduction misalignment, it seems reasonable to think that lack of alignment is a problem. From a practical and policy perspective, alignment among what components are offered, used, and are helpful would be ideal in terms of meeting veteran needs. It is possible that alignment improves over time; however, this assertion has yet to be examined.

For new post-9/11veterans, tangible support components were the most widely used, and this was particularly true for VA programs. Three-quarters of new veterans reported using at least one VA program. VA benefits are a unique tangible support component because they are offered at no cost and directly enable a person to obtain a desired outcome (i.e., higher education or home ownership). VA-sponsored education was used by 43% of the sample. This assistance enables veterans or their family members to achieve a higher level of education. Education is, of course, positively associated with a host of health and well-being outcomes (e.g., higher paying jobs, lower morbidity). The VA home loan benefit was used by 32% of veterans. Veterans whose military occupation was combat arms and combat support were more likely to utilize educational benefits because their military occupations may not translate directly to civilian occupations; thus, additional training could be needed to find employment within the civilian population.

Three additional commonly reported tangible support barrier reduction components were directly related to meeting a veteran’s economic needs (i.e., cash, clothing and consumer goods, food, and discounted pricing on goods and services). A subset of post-9/11 veterans struggle financially, and this is particularly true for veterans who have health problems [42], live in poverty [43], and are female [44]. More community-based organizations should consider focusing on providing for the basic needs of this subset of veterans and their families.

In comparison, very few veterans (16%) utilized the access barrier reduction components for non-VA benefits (e.g., Tricare, Medicare, Army Wounded Warrior Program). One potential reason may be that veterans may not be eligible for all barrier reduction components available within a program. It is also possible that veterans are unaware of the resources available to them when using a program. For example, income guidelines or minimum credit scores may need to be met before a veteran can access certain benefits.

The barrier reduction component of access assistance was also found to be a helpful tool that veterans used. For instance, approximately 33% of veterans report utilizing at least one VA healthcare service (i.e., hospital, clinic, or Vet Center). Moreover, as consistent with previous research [32], veterans with a medical discharge were 24% more likely to use VA healthcare services. In addition, veterans with ongoing physical health conditions were two times more likely to utilize VA healthcare, and those with mental health problems were 58% more likely to use VA healthcare compared to those without physical/ mental health conditions. Access to VA benefits is a primary concern for veterans as they transition to civilian life [17]. Further research should explore which specific VA benefits are the most challenging to navigate, and develop strategies to help veterans overcome these challenges.

The majority of programs were found to make access easier by making some of their materials available online (97% of programs). For example, the VA is widening its access to veterans by providing more administrative support and clinical care via the web. Implementation science theory and research supports the idea of making access to information and resources more widely available. However, navigating the internet to find the specific program may still be troublesome for some veterans. In this ever advancing technological world, web access will play an increasingly important role in the lives of veterans; however, rural dwelling and older veterans often have poor or no access to the internet [45]. Thus, policies that extend the reach of internet access to rural areas could close the gap in web access.

Transportation appears to be a key access assistance component particularly for veterans with serious injuries or disabilities that prevent them from getting around by themselves or for those who do not have the financial means to purchase and maintain their own transportation [46]. However, while 20% of veterans nominated programs that offer transportation, only 2.7% reported using and benefiting from the component. Transportation for subsets of veterans may still be important, and these subsets should be identified. In this study, veterans who screened positive for PTSD symptoms were two times more likely to utilize programs with a transportation component. On the other hand, veterans with physical health conditions were not significantly more likely to utilize programs that provide transportation. As a result, future research should attempt to understand which veterans will value and benefit from a transportation component. In so doing, program developers will be able to incorporate the provision of transportation as part of their program’s portfolio of support in a manner that matches veteran needs.

Access to child care can be a barrier reduction component as it provides service members free time to utilize programs or pursue employment or educational opportunities [47]. However, child care was rarely mentioned by veterans as a barrier reduction component that programs offered. Prior research has shown that veterans report having limited access to child care services [48]. Moreover, male and female veterans report that the VA should offer child care services and, if these services were offered, they would use them [48]. Several studies with civilian families demonstrate that the provisions of child care and meals are inducements to program participation, particularly for families who experience financial and other hardships [49–51]. The provisions of child care and meals to enhance program participation, while primarily used in prevention research studies in university-community based partnerships, are barrier reduction components that may be transportable to other community-based organizations that offer programs to veterans and their families.

The barrier reduction components related to intra-individual change (i.e., focus on increasing motivation to change and stigma reduction) were present in a low proportion of programs. However, these components were used and viewed as helpful by 17.5% of veterans. Among those programs that offered this component, 29.3% of veterans reported using it. Military and veteran cultures foster norms that stigmatize help seeking, particularly for mental health problems [52]. Active duty military and veterans also express a significant degree of distrust of institutions designed to support them [19]. Senior enlisted and officers were more likely to utilize programs with a stigma reduction component compared to junior enlisted (E1 to E4). Veterans with a mental health condition were two times more likely to utilize programs with a stigma reduction component. Greater focus on intra-individual change components makes sense. For example, approaches designed to reorient help-seeking norms of military and veterans to be more open to help seeking and more accepting of people’s health challenges have shown promise [10]. Several attempts at stigma reduction approaches have been developed for the military and veteran contexts [35, 36, 52], however, they have yet to be evaluated for effectiveness. Veteran serving organizations should investigate the feasibility of adopting these approaches.

As with any study, there were some limitations with the current investigation. The most significant limitation of the study is that the sample was not drawn at random. As a result, the degree to which the findings were biased is not known. Also, participation in the study was voluntary and it is likely that respondents who participated felt strongly about the topic in question and may favor certain responses [53]. While the cell weighting used in this study statistically adjusted the sample to better reflect the three characteristics that were known of the population (i.e., gender, paygrade, branch), it does not yield the strength of inference that can be drawn from random sampling. Nevertheless, the opportunity to participate was offered to the population of recently separated post 9–11 veterans during the period of June–September 2016. Moreover, a variety of methods to recruit veterans were used (e.g., phone, paper version, online survey). Thus, every veteran had an equal probability of being invited to participate. Details on the sampling methodology have been previously reported (see Vogt, et al., 2018) and interested readers may consider reading this manuscript. Future studies should consider drawing additional samples of new post-9/11 veterans to compare them to the results of this study. A nationally representative sample of new post-9/11 veterans would yield unbiased estimates of their perceptions of barrier reduction techniques. Second, veterans were likely to use more programs than they described in detail because the survey limited in-depth self-report information to two programs. Third, the analysis of web-based coding may be biased because it is likely that there were at least some differences between what was offered to veterans and the veterans’ own perception of what was offered. Nonetheless, what seems most important is whether or not veterans perceived the program components as helpful or not. Future qualitative work is recommended to elucidate veteran perceptions of specific program components. Fourth, this study examined the first 3 months after separation from the military. Subsequent analyses from TVMI will examine how the perceptions of barrier reduction strategies change over time.

## Conclusions

To expand the reach and penetration of programs and services to post-9/11 veterans, barrier reduction efforts should be robust and align with what veterans report valuing as they transition from active duty to civilian life. Currently, with the exception of tangible supports, the barrier reduction efforts that veterans appear to value (e.g., assistance in gaining access to support resources) are rarely offered and they are under-utilized by veterans. Veterans who are at most risk for poor civilian reintegration are also least likely to engage programs that offer barrier reduction components. Veterans serving organizations should consider bolstering their efforts at providing barrier reduction efforts, particularly with respect to better alignment with veterans’ needs.

## Data Availability

NRM had full access to all the data and takes responsibility for the integrity of the data and accuracy of the data analysis. The datasets generated and/or analyzed during the current study are not currently publicly available due to the ongoing collection of data in The Veterans Metrics Initiative. However, the data will be publicly available in April of 2021. The data and will be hosted by and will be hosted by ICPSR which is a unit within the Institute for Social Research at the University of Michigan. Parties interested in accessing the data described in this study should contact Keith R. Aronson, Ph.D., Associate Director, Clearinghouse for Military Family Readiness at Penn State University, 114 J Henderson Building, University Park, PA 16802, kra105@psu.edu, (814)-865–6909.

## Abbreviations

PTSD: Posttraumatic Stress Disorder
TVMI: The Veterans Metrics Initiative
VA: U.S. Department of Veterans Administration
VADIR: Veterans Affairs and Department of Defense Identity Repository
VHA: Veterans Health Administration
